# Prevalence and clinical characteristics of carotid atherosclerosis in newly diagnosed patients with ketosis-onset diabetes: a cross-sectional study

**DOI:** 10.1186/1475-2840-12-18

**Published:** 2013-01-16

**Authors:** Lian-Xi Li, Cui-Chun Zhao, Ying Ren, Yin-Fang Tu, Jun-Xi Lu, Xing Wu, Wei-Xing Zhang, Jia-An Zhu, Mei-Fang Li, Li-Bo Yu, Yu-Qian Bao, Wei-Ping Jia

**Affiliations:** 1 Department of Endocrinology and Metabolism, Shanghai Jiao Tong University Affiliated Sixth People’s Hospital; Shanghai Diabetes Institute, Shanghai Clinical Center for Diabetes; Shanghai key Laboratory of Diabetes Mellitus, 600 Yishan Road, Shanghai, 200233, China; 2 Department of VIP, Shanghai Jiao Tong University Affiliated Sixth People’s Hospital, 600 Yishan Road, Shanghai, 200233, China; 3 Department of Ultrasonography, Shanghai Jiao Tong University Affiliated Sixth People’s Hospital, 600 Yishan Road, Shanghai, 200233, China

**Keywords:** Ketosis-prone diabetes, Type 2 diabetes, Atherosclerosis, Carotid arteries, Epidemiology

## Abstract

**Background:**

The features of carotid atherosclerosis in ketosis-onset diabetes have not been investigated. Our aim was to evaluate the prevalence and clinical characteristics of carotid atherosclerosis in newly diagnosed Chinese diabetic patients with ketosis but without islet-associated autoantibodies.

**Methods:**

In total, 423 newly diagnosed Chinese patients with diabetes including 208 ketosis-onset diabetics without islet-associated autoantibodies, 215 non-ketotic type 2 diabetics and 79 control subjects without diabetes were studied. Carotid atherosclerosis was defined as the presence of atherosclerotic plaques in any of the carotid vessel segments. Carotid intima-media thickness (CIMT), carotid atherosclerotic plaque formation and stenosis were assessed and compared among the three groups based on Doppler ultrasound examination. The clinical features of carotid atherosclerotic lesions were analysed, and the risk factors associated with carotid atherosclerosis were evaluated using binary logistic regression in patients with diabetes.

**Results:**

The prevalence of carotid atherosclerosis was significantly higher in the ketosis-onset diabetic group (30.80%) than in the control group (15.2%, p=0.020) after adjusting for age- and sex-related differences, but no significant difference was observed in comparison to the non-ketotic diabetic group (35.8%, p=0.487). The mean CIMT of the ketosis-onset diabetics (0.70±0.20 mm) was markedly higher than that of the control subjects (0.57±0.08 mm, p<0.001), but no significant difference was found compared with the non-ketotic type 2 diabetics (0.73±0.19 mm, p=0.582) after controlling for differences in age and sex. In both the ketosis-onset and the non-ketotic diabetes, the prevalence of carotid atherosclerosis was markedly increased with age (both p<0.001) after controlling for sex, but no sex difference was observed (p=0.479 and p=0.707, respectively) after controlling for age. In the ketosis-onset diabetics, the presence of carotid atherosclerosis was significantly associated with age, hypertension, low-density lipoprotein cholesterol and mean CIMT.

**Conclusions:**

The prevalence and risk of carotid atherosclerosis were significantly higher in the ketosis-onset diabetics than in the control subjects but similar to that in the non-ketotic type 2 diabetics. The characteristics of carotid atherosclerotic lesions in the ketosis-onset diabetics resembled those in the non-ketotic type 2 diabetics. Our findings support the classification of ketosis-onset diabetes as a subtype of type 2 diabetes.

## Background

In recent decades, a ketosis-prone form of diabetes that does not necessarily fit the typical characteristics of autoimmune type 1 diabetes mellitus has been identified. Because the aetiology of this form of diabetes is unclear and the classic markers of autoimmune destruction of islet beta cells are also absent, the World Health Organization and the American Diabetes Association have classified this subtype as “idiopathic Type 1 diabetes” or “Type 1B” [1]. However, growing evidence, including older age at onset, higher rates of obesity and genetic predisposition among affected individuals, suggest that this atypical form of diabetes should be considered as a subset of type 2 diabetes, or ketosis-prone type 2 diabetes [2,3].

Although the metabolic and autoimmune characteristics of ketosis-prone type 2 diabetes have been well documented [3-6], little is known concerning the prevalence and clinical features of atherosclerosis in ketosis-prone type 2 diabetics. It is well-established that diabetes mellitus is an independent risk factor for atherosclerosis and cardiovascular disease [7-10]. Several studies have demonstrated that patients with diabetes are at higher risk for atherosclerosis and cardiovascular disease in comparison to normal subjects [11-15]. According to our previous study, the prevalence of atherosclerosis detected by ultrasound examination was nearly 80% in hospitalised type 2 diabetics [16]. However, few studies have investigated the prevalence and clinical characteristics of atherosclerosis in patients with ketosis-prone type 2 diabetes.

Thus, one of the aims of the present study was to investigate the prevalence of carotid atherosclerosis in newly diagnosed Chinese patients with diabetes compared with control individuals. Secondly, our study aimed to investigate differences in carotid atherosclerosis and compare clinical, anthropometric and metabolic differences between the ketosis-onset and the non-ketotic type 2 diabetics. Finally, we aimed to evaluate the clinical features of carotid atherosclerosis and to determine the risk factors for carotid atherosclerosis in both ketosis-onset and non-ketotic diabetic patients.

## Materials and methods

### Subjects and study design

This was a cross-sectional study and was partly based on data obtained in our previous study [16]. Between January 2007 and October 2008, 497 Chinese patients (≥17 years old) newly diagnosed with diabetes were hospitalised in our department. According to the criteria of the World Health Organization [1], newly diagnosed diabetes was defined as having either fasting plasma glucose (FPG) ≥ 7.0 mmol/L and/or 2-h postprandial plasma glucose (2-h PPG) ≥ 11.1 mmol/L in subjects without a history of diabetes. Diabetic ketosis was defined as the presence of hyperglycaemia and moderate to heavy urine ketones (0.5 mmol/L-15 mmol/L). Patients with concomitant conditions that might result in ketosis were excluded. These conditions included gestational diabetes, renal insufficiency, corticoid therapy, and infectious disease. Ketosis-onset diabetes was defined as diabetes with the presence of diabetic ketosis and in the absence of glutamic acid decarboxylase (GAD) and thyrosin phosphatase (IA-2) autoantibodies. Non-ketotic type 2 diabetes was defined as diabetes with neither diabetic ketosis nor GAD or IA-2 autoantibodies. Of these 497 patients, 55 patients who were positive for islet-associated autoantibodies were excluded, and 19 patients who did not meet other criteria were also excluded. The other 423 patients with diabetes were classified into two categorical groups based on the presence or absence of diabetic ketosis when diagnosed with diabetes. Of these patients, 208 were diagnosed for ketosis-onset diabetes and 215 were diagnosed for non-ketotic type 2 diabetes. In addition, 79 control participants with a FPG < 6.0 mmol/L and a 2-h plasma glucose < 7.8 mmol/L during a 75 g oral glucose tolerance test were age-matched to the patients with ketosis-onset diabetes.

All subjects were interviewed to obtain their history of hypertension, alcohol consumption and smoking habits. Smoking included both current and former smokers. Likewise, alcohol use included current and former use of alcohol. The study was approved by the human research ethics committee of the hospital, and informed consent was obtained from all subjects.

### Physical examination and laboratory measurements

Physical examinations measuring weight, height, waist circumference, hip circumference and blood pressure were performed by our previous method [16]. Body mass index (BMI) was calculated as weight divided by height squared. The waist-to-hip ratio (WHR) was calculated as waist circumference divided by hip circumference. Underweight was defined as a BMI of less than 18.5 kg/m^2^, overweight as 23 to 24.9 kg/m^2^, and obesity as above 25 kg/m^2^ based on the Asia-Pacific criteria set by the World Health Organization [17]. Hypertension was defined according to our previously described criteria [16].

All patients with diabetes were screened for IA-2 and GAD autoantibodies at the time of admission. The autoantibodies against GAD and IA-2 were measured by ELISA (EUROIMMUN Medizinische Labordiagnostika AG, Germany). Urine ketones were measured by Legal’s test.

Venous blood samples were collected after an overnight fast and 2 h after breakfast. Fasting Glycosylated hemoglobin A1C (HbA1c), fasting C-peptide (FCP), 2-h postprandial C-peptide (2-h PCP), fasting plasma glucose (FPG), 2-h postprandial plasma glucose (2-h PPG), total triglycerides (TG), total cholesterol (TC), high-density lipoprotein cholesterol (HDL-C), and low-density lipoprotein cholesterol (LDL-C) were measured by standard laboratory methods.

### Ultrasonography measurements

Each subject was submitted to carotid artery Doppler ultrasound examination. Carotid ultrasonography was performed by three experienced ultra-sonographers under a standardised protocol. Colour Doppler sonography was performed with an Acuson Sequoia 512 scanner (Siemens Medical Solutions, Mountain View, CA) equipped with a 5–13 MHz linear array transducer.

All subjects were examined in a supine position with their head turned 45 degrees from the site being scanned. Carotid arteries were examined bilaterally at the common carotid arteries, the bifurcation, the external carotid arteries, and the internal carotid arteries from transverse and longitudinal orientations and were scanned in the anterolateral, posterolateral and mediolateral directions to assess the presence of atherosclerotic plaque and stenosis and measure CIMT. Measurements were made manually on still images magnified to standard size on-line. The IMT was defined as the distance between the leading edge of the lumen-intima echo and the leading edge of the media-adventitia echo. The mean CIMT was defined as the mean of the right and left IMT of the common carotid artery. The common carotid artery IMT was measured on-line in the posterior wall 10–20 mm proximal to the carotid bifurcation in a region free of focal plaque. Three measurements were made on each side, and the values were averaged to produce a mean IMT for each side. According to the Mannheim consensus [18], atherosclerotic plaques were defined as focal structures encroaching into the arterial lumen of 0.5 mm or 50% of the surrounding IMT value or IMT of > 1.5 mm. Carotid atherosclerosis was defined as the presence of atherosclerotic plaques in any of the aforementioned arterial segments. Carotid stenosis was defined as any degree of narrowing in either the internal, external or common carotid arteries by carotid plaques. Both intra-observer and inter-observer reproducibility were determined using a Spearman correlation coefficient. The intra-observer correlation coefficients were 0.91-0.92 for carotid IMT, 0.88-0.89 for the identification of carotid plaques, and 0.87-0.94 for the identification of carotid stenosis. The inter-observer correlation coefficients were 0.87-0.97 for carotid IMT, 0.83-0.94 for the identification of carotid plaques, and 0.82-0.89 for the identification of carotid stenosis.

### Statistical analyses

The SPSS 11.0 software was used for statistical analysis. A p-value less than 0.05 was considered as statistically significant. Data are presented as the mean ± S.D., and median values are provided for skewed data. Contingency table analyses with chi-square tests were used to assess differences in the relative frequencies of categorical variables. Binary or multinomial logistic regression was applied to assess differences of categorical variables while adjusting for age and/or sex. Normality was checked for continuous variables. If the data were distributed normally, one-way ANOVA with LSD was used to compare quantitative data among three groups, and independent sample t tests were used for comparisons of continuous variables between two groups. The Mann–Whitney *U* test was used if the data were not distributed normally. Linear regression was used to evaluate differences of continuous variables while adjusting for age and/or sex. The Mantel-Haenszel Common Odds Ratio Estimate was used to assess the odds ratio of carotid atherosclerosis while controlling for the effects of age and sex. Binary logistic regression analysis was performed to investigate the associations between risk factors for atherosclerosis and carotid atherosclerosis.

## Results

### Characteristics of study subjects

The clinical characteristics of the studied subjects are presented in Table 1. Both the control group and the non-ketotic type 2 diabetic group had an equal sex distribution, whereas the ketosis-onset diabetic group had a strong predominance of males, even after adjusting for age. The ketosis-onset diabetic patients had significantly higher FPG, 2-h PPG and HbA1c levels than the non-ketotic type 2 diabetic patients (all p<0.001). The levels of FCP and 2-h PCP were markedly lower in the ketosis-onset diabetic patients than in the non-ketotic diabetic patients before and after adjusting for age and sex (all p<0.001).

**Table 1:** Characteristics of study subjects

**Variables**	**Control subjects**	**Ketosis-onset type 2 diabetes**	**Non-ketotic type 2 diabetes**	**P value**	*P value	^**#**^**P value**
	**(n=79)**	**(n=208)**	**(n=215)**			
Male (%)	46 (58.2%)	153 (73.6%)	122 (56.7%)	0.001	0.009	0.01
Age (years)	50±7	49±15	56±14	<0.001	<0.001	<0.001
Smoking (n, %)	44 (55.7%)	84 (40.4%)	65 (30.2%)	<0.001	<0.001	0.740
Alcohol (n, %)	44 (55.7%)	37 (17.8%)	36 (16.7%)	<0.001	<0.001	0.311
Hypertension (%)	17 (21.5%)	81 (38.9%)	98 (45.6%)	0.001	0.026	0.685
BMI (kg/m2)	24.64±3.5	25.05±3.54	25.16±3.59	0.548	0.095	0.251
WHR	0.90±0.05	0.91±0.06	0.91±0.06	0.051	0.128	0.894
Weight category				0.648	0.365	0.631
underweight	0 (0.00%)	3 (1.4%)	2 (0.9%)	——	——	——
overweight	23 (29.1%)	48 (23.1%)	48 (22.3%)	——	——	——
obesity	32 (40.5%)	97 (46.6%)	109 (50.7%)	——	——	——
SBP (mmHg)	129±14	127±15	130±17	0.162	0.907	0.523
DBP (mmHg)	84±12	81±11	81±9	0.039	0.073	0.729
*TG (mmol/l)	1.52 (1.05-2.04)	1.44 (1.00-2.19)	1.49 (1.08-2.21)	0.711	0.394	0.831
TC (mmol/l)	5.05±1.10	5.03±1.22	4.82±1.22	0.163	0.080	0.035
HDL (mmol/l)	1.45±0.34	1.05±0.27	1.11±0.25	<0.001	<0.001	0.390
LDL (mmol/l)	3.78±1.24	3.40±1.03	3.12±0.97	<0.001	<0.001	0.002
*FPG (mmol/l)	5.36 (5.00-5.66)	9.74 (7.63-12.45)	7.53 (6.39-9.67)	<0.001	<0.001	<0.001
*2h PPG (mmol/l)	5.00 (4.48-6.08)	16.66 (12.94-20.79)	13.14 (10.39-16.66)	<0.001	<0.001	<0.001
*FCP	——	1.23 (0.74-1.92)	2.02 (1.36-2.93)	<0.001	<0.001	<0.001
*2h PCP	——	2.31 (1.55-3.66)	4.65 (3.12-6.35)	<0.001	<0.001	<0.001
HA1C (%)	5.44±0.43	11.83±2.05	9.81±2.58	<0.001	<0.001	<0.001

Values are expressed as the mean±S.D, median with interquartile range, or percentages. * Non-normal distribution of continuous variables.

P-value: The p-values were not adjusted for age and sex for the trend.

*P-value: The *p-values were adjusted for age and sex for the trend.

^#^P-value: The ^#^p-values were adjusted for age and sex for ketosis-onset type 2 diabetes vs.non-ketotic type 2 diabetes.

### Comparison of carotid atherosclerosis

**Figure 1:**
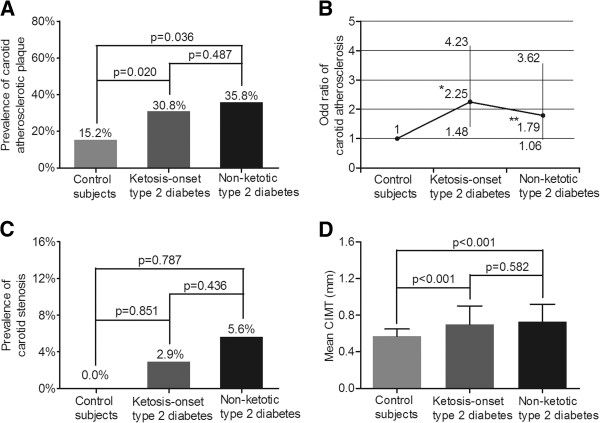
**Comparison of carotid atherosclerotic lesions among the three groups after adjusting for age and sex. **(**A**) Comparison of the prevalence of carotid atherosclerosis among the three groups. The p-value for three group comparisons was 0.045. (**B**) Odds ratio of carotid atherosclerosis for the ketosis-onset and the non-ketotic type 2 diabetic subjects in comparison to control subjects without diabetes. The bars represent the 95% confidence interval. Compared with control subjects, where *p=0.024 and **p=0.041. (**C**) Comparison of the prevalence of carotid atherosclerotic stenosis among the three groups. The p-value for three group comparisons was 0.738 (**D**) Comparison of mean CIMT among the three groups. The p-value for three group comparisons was <0.001.

The comparison of carotid atherosclerosis among the control group, the ketosis-onset diabetic group and non-ketotic diabetic group after adjustment for age and sex is shown in Figure 1. The prevalence of carotid atherosclerosis in the ketosis-onset diabetic group was significantly higher than in the control group (p=0.020), but no significant difference was observed between the ketosis onset and non-ketotic type 2 diabetic groups (p=0.487) (Figure 1A). The odds ratio of carotid atherosclerosis was higher in the patients with ketosis-onset diabetes than in the control group (OR, 2.25; 95% CI, 1.48-4.23; P=0.024), but the odds ratio of carotid atherosclerosis between ketosis-onset and non-ketotic diabetics did not differ significantly (Figure 1B). In contrast to carotid atherosclerosis, there was no significant difference in the prevalence of carotid stenosis among the control group (0.0%), the ketosis-onset diabetic group (2.9%) and non-ketotic diabetic group (5.6%) (p=0.738) (Figure 1C). Similar to carotid atherosclerosis, the mean CIMT in the ketosis-onset diabetics (0.70±0.20 mm) was significantly greater than that of the control subjects (0.57±0.08 mm, p<0.001), but no significant difference was found in comparison to the non-ketotic type 2 diabetic subjects (0.73±0.19 mm, p=0.582) (Figure 1D).

### Comparison of clinical characteristics between the subjects with and without carotid atherosclerosis

The clinical and laboratory characteristics of the studied subjects with and without carotid atherosclerosis in each group are shown in supplementary data (Additional file 1: Table S1). In the ketosis-onset diabetic group, variables that were significantly different between the subjects with and without carotid atherosclerosis included age, hypertension, SBP, LDL-C, and FPG. Interestingly, similar to the ketosis-onset diabetic group, variables in the non-ketotic type 2 diabetic group that were significantly different between the subjects with and without carotid atherosclerosis also included age, hypertension, and SBP.

### Comparison of mean CIMT between the subjects with and without carotid atherosclerosis

**Figure 2:**
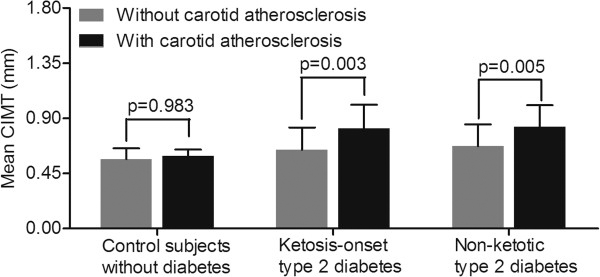
Comparison of mean CIMT between subjects with and without carotid atherosclerosis.

Figure 2 compares the mean CIMT value of subjects with and without carotid atherosclerosis in each group. In both the ketosis-onset (0.64±0.18 mm and 0.82±0.19 mm for the subjects without and with carotid atherosclerosis, respectively, p=0.003) and non-ketotic (0.67±0.18 mm and 0.83±0.18 mm for the subjects without and with carotid atherosclerosis, respectively, p=0.005) diabetic groups, the mean CIMT value was markedly higher in the subjects with carotid atherosclerosis than in the subjects without carotid atherosclerosis.

### Analyses of carotid atherosclerotic lesions and risk factors for carotid atherosclerosis in type 2 diabetes

**Figure 3:**
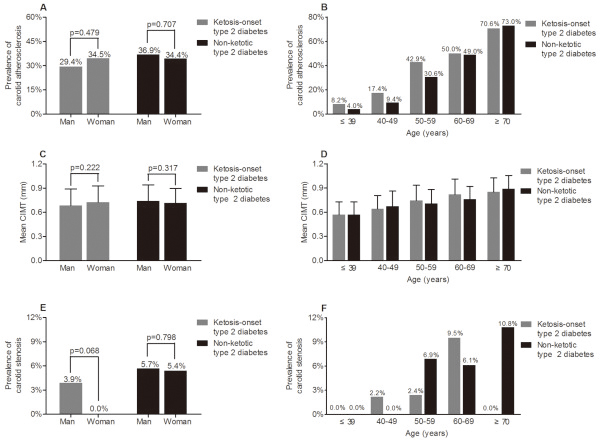
**Comparison of carotid atherosclerotic lesions stratified by sex and age in type 2 diabetics. **(**A**) The prevalence of carotid atherosclerosis stratified by sex in ketosis-onset and non-ketotic type 2 diabetic subjects after adjusting for age. (**B**) The prevalence of carotid atherosclerosis stratified by age in ketosis-onset and non-ketotic type 2 diabetic subjects after adjusting for sex. The p-values for group comparisons were all <0.001 in both the ketosis-onset and the non-ketotic type 2 diabetic subjects. (**C**) Comparison of mean CIMT stratified by sex in ketosis-onset and non-ketotic type 2 diabetic subjects after adjusting for age. (**D**) Comparison of mean CIMT stratified by age in ketosis-onset and the non-ketotic type 2 diabetic subjects after adjusting for sex. The p-values for group comparisons were all <0.001 in both the ketosis-onset and the non-ketotic type 2 diabetics. (**E**) The prevalence of carotid atherosclerotic stenosis stratified by sex in ketosis-onset and non-ketotic type 2 diabetic subjects after adjusting for age. (**F**) The prevalence of carotid atherosclerotic stenosis stratified by age in ketosis-onset and non-ketotic type 2 diabetic subjects after adjusting for sex. The p-values for group comparisons were 0.560 and 0.626 in the ketosis-onset and the non-ketotic type 2 diabetics, respectively.

The analyses of carotid atherosclerotic lesions with respect to sex and age in type 2 diabetes are shown in Figure 3. There was no significant difference in the mean CIMT and the prevalence of carotid atherosclerotic plaques and stenosis in patients with diabetes between the sexes (Figure 3A, C and E). To the contrary, the mean CIMT and the prevalence of carotid atherosclerotic plaques significantly increased with age in diabetic patients (Figure 3B and D). However, there was no significant age-related difference in the prevalence of carotid stenosis in diabetic patients (Figure 3F).

A multivariate analysis of variables associated with carotid atherosclerosis in the diabetic patients is shown in Table 2. According to the analyses, statistically significant risk factors for carotid atherosclerosis were age, hypertension, LDL-C, and mean CIMT in the ketosis-onset diabetics, and age, hypertension and mean CIMT were risk factors in the non-ketotic diabetics. For all type 2 diabetics studied, diabetic ketosis was not a risk factor for carotid atherosclerosis.

**Table 2:** A multivariate analysis of variables associated with carotid atherosclerosis

**Variables**	**Ketosis-onset type 2 diabetes**			**Non-ketotic type 2 diabetes**			**Total type 2 diabetes**		
	**OR**	**95% CI**	**p value**	**OR**	**95% CI**	**p value**	**OR**	**95% CI**	**p value**
Sex	1.25	0.43-3.67	0.679	2.39	0.79-7.27	0.124	1.73	0.83-3.59	0.145
Age	1.05	1.01-1.09	0.006	1.10	1.05-1.15	<0.001	1.07	1.04-1.10	<0.001
Hypertension	3.00	1.12-8.02	0.029	1.34	1.81-5.53	0.045	1.98	1.57-6.85	0.037
LDL-C	1.56	1.01-2.40	0.046	1.23	0.77-1.98	0.392	1.37	1.01-1.86	0.045
Mean CIMT	9.73	1.08-87.55	0.042	19.42	2.69-94.11	0.007	16.18	3.14-83.44	0.001
Diabetic ketosis							0.95	0.50-1.80	0.881

Binary logistic regression analyses were performed with the presence of carotid atherosclerosis as the dependent variable and with risk factors including age, sex, hypertension, SBP, BMI, HDL-C, LDL-C, FPG, FCP, HA1C, mean CIMT, and diabetic ketosis as independent variables. The table only includes sex and variables significantly associated with carotid atherosclerosis.

## Discussion

Atypical or ketosis-prone type 2 diabetes was first reported by Winter *et al.* in 1987 in black Americans [19] and is distinguishable from other subtypes of diabetes by clinical, immunological, and biological features. It has since been reported in other ethnicities such as Native-Americans, Chinese, and Hispanics [20-22]. Atypical ketosis-prone diabetes is frequently detected in obese individuals and characterised by an acute onset with either ketosis or ketoacidosis [6,23]. In addition, patients with atypical ketosis-prone diabetes lack markers associated with islet cell autoimmunity.

The clinical, metabolic, and immunological characteristics of atypical ketosis-prone diabetes have been well studied by other investigators [4,21,22,24]. However, controversy exists concerning the classification of atypical ketosis-prone diabetes because the disease possesses features associated with both type 1 and type 2 diabetes. Atypical ketosis-prone diabetes was previously classified as idiopathic Type 1 diabetes by the World Health Organization and the American Diabetes Association [1]. On the other hand, some researchers think that atypical ketosis-prone diabetes is a subtype of type 2 diabetes that develops in patients with high sensitivity to glucotoxicity or lipotoxicity or with dysregulated glucagon secretion [2,3,6]. Furthermore, the prevalence and features of atherosclerosis in both type 1 and type 2 diabetes are well-documented, but those in patients with ketosis-prone type 2 diabetes remain unknown and have not been investigated. Thus, in the current study we focused our attention on investigating the prevalence and clinical characteristics of carotid atherosclerosis in patients with diabetic ketosis but without islet autoantibodies at onset. To the best of our knowledge, this is the first report to describe the prevalence and clinical characteristics of atherosclerosis in ketosis-onset type 2 diabetes.

### General clinical features

Because our subjects were negative for islet-associated autoantibodies, the relationship between ketosis-onset and a transient dysfunction of islet cells through an autoimmune process was considered to be unlikely in our studied cohorts. Therefore, we proposed two distinct diabetic groups based on presence or absence of diabetic ketosis. The prevalence of ketosis-prone type 2 diabetes is not known, but our findings indicated that what was once described as “atypical diabetes” is in fact a common clinical phenomenon affecting nearly 50% of Chinese patients with newly diagnosed diabetes in our study. The reason that nearly half of newly diagnosed diabetic subjects had ketosis-onset is that the condition of ketosis-onset diabetic subjects tended to be more serious, and these patients were thus more likely to be hospitalised.

The patients with ketosis-onset diabetes showed an earlier onset, with a mean age of 49 years considering that the mean age of the patients with non-ketotic type 2 diabetes was 56 years old. Most importantly, unlike cases of typical type 1 and type 2 diabetes, which display a similar sex distribution, ketosis-onset diabetes shows a stronger prevalence in males consistently with previous reports [5,25-27]. Additionally, consistent with several previous studies [25,26], we also found that there were higher blood glucose levels and lower plasma C-peptide levels in ketosis-onset diabetics compared with the non-ketotic type 2 diabetics, which indicated the insulin secretion function of islets was impaired at onset in the patients with ketosis-prone diabetes. The acute impairment of β-cell function in ketosis-prone diabetes has been found to be the major determinant of ketosis onset [24]. Although the underlying mechanisms of impaired islet β-cell function have not been elucidated, an increased susceptibility to glucose toxicity or lipotoxicity might result in the transient functional abnormalities of islet β-cells [25,27,28].

On the other hand, there were many similarities in the clinical characteristics between the ketosis-onset diabetics and the non-ketotic type 2 diabetics. For example, the proportion of overweight and obesity in the ketosis-onset diabetics was similar to that of the non-ketotic type 2 diabetics. Obesity was present in 46.6% of the ketosis-onset diabetic patients in our study, which was very close to the findings of Winter and colleagues [19]. Additionally, both BMI and WHR were also similar between the two groups, which indicated that individuals with ketosis-prone diabetes physically resemble those with type 2 diabetes. Likewise, we observed older age, a higher proportion of hypertension, higher SBP, and a greater mean CIMT value in subjects with carotid atherosclerosis than in those without carotid atherosclerosis, and this was shared by between ketosis-onset and non-ketotic type 2 diabetic groups.

### Carotid atherosclerotic lesions

Previous studies have described the prevalence of carotid atherosclerosis in both type 1 and type 2 diabetes, but there are very few reports available for ketosis-prone type 2 diabetes. Thus, we further evaluated the prevalence and clinical features of carotid atherosclerosis in ketosis-onset diabetes. Our results demonstrated a significantly increased prevalence of carotid atherosclerosis in newly diagnosed diabetic patients with ketosis-onset compared with the control subjects without diabetes but not in comparison to the non-ketotic diabetic patients. Moreover, the diabetic patients with ketosis-onset had a nearly 2.3-fold increased risk of carotid atherosclerosis in comparison to the control subjects consistent with findings of other studies of typical type 2 diabetic patients. On the contrary, when compared with the non-ketotic type 2 diabetes, the risk of carotid atherosclerosis was not markedly increased or decreased in the ketosis-onset diabetes. In a population-based study, carotid atherosclerosis was present in 58% of the study subjects [29]. A study by Lundman *et al.*[30] showed that 28% of type 2 diabetic patients had one or more carotid plaques. After a 2-year follow-up, the prevalence of carotid plaques increased to 62%, including patients who initially had plaques. It is well-established that carotid artery plaques are markers of systemic subclinical atherosclerosis and strong predictors of cardiovascular events [29,31,32]. A large population-based study has demonstrated a significant association between the presence of carotid plaques and the risk of vascular events [32]. Similar to other studies [33-35], the prevalence of carotid atherosclerosis also significantly increased with age in the patients with ketosis-onset diabetes, but there was no significant difference between sexes in our study which may be due to uneven gender distribution in diabetic patients with ketosis-onset [5,25,27]. Given that carotid atherosclerosis is associated with an increased risk of cardiovascular disease and is also a powerful predictor for future cardiovascular events, the presence of carotid atherosclerosis in ketosis-prone diabetic patients may contribute to the improved prediction of the future risk of vascular events.

In contrast to carotid atherosclerosis, the prevalence of carotid stenosis was low in both newly diagnosed ketosis-onset diabetics and non-ketotic type 2 diabetics, and no significant difference was noted among the three groups studied. The estimated prevalence of carotid artery stenosis varies to a great extent in different studies and is dependent on the definition of carotid stenosis, the method for measuring stenosis, and the selected study populations [13,36,37]. In the Tromsø Study, the prevalence of carotid stenosis was 5.3% in men and 3.8% in women in the general population [36]. Lacroix *et al.* demonstrated that the prevalence of carotid stenosis <60% and ≥ 60% was 63.6% and 4.7% in type 2 diabetic patients without any history of cerebrovascular disease, respectively [37], which was far higher than that of our study subjects. This discrepancy was partly explained by the fact that our study subjects were newly diagnosed with diabetes although atherosclerotic stenosis is more frequently detected in the diabetic patients with a long history of diabetes. Furthermore, the prevalence of carotid stenosis is significantly higher in men than in women and increases with age as shown in other studies [36,37]. In our study, although the prevalence of carotid stenosis stratified by sex and age was not statistically significant different in the patients with ketosis-onset diabetes, the prevalence of carotid stenosis was also higher in men (3.9%) than in women (0.0%). Likewise, the prevalence of carotid stenosis increased with age in patients with ketosis-onset diabetes. Given that ketosis-onset diabetes has a strong male predominance, the above results may be due to small samples and uneven gender distribution in the ketosis-onset diabetic group. Carotid stenosis is one of the main causes of cerebrovascular events, and the degree of carotid stenosis is also a valid marker of the risk of cerebrovascular events. Therefore, the presence of carotid artery stenosis can be used as an indicator to select patients for interventional procedures among ketosis-prone diabetics, especially in asymptomatic subjects.

Similarly to carotid atherosclerosis, the mean CIMT value showed a significant increase in the ketosis-onset diabetic subjects in comparison to the control subjects. Additionally, in ketosis-onset diabetes the mean CIMT value was directly related to the age of the patients, and the elderly patients had a higher mean CIMT value compared with younger patients consistent with findings made by previous investigators [16,38-41]. Interestingly, we could not find gender differences in CIMT values in the ketosis-onset diabetic patients, and this was in accordance with a previous study in children with type 1 diabetes mellitus [40]. We speculate that it may also be due to small sample size and uneven gender distribution in the ketosis-onset diabetic group. The measurement of the IMT of carotid arteries assessed by ultrasound is often used to detect early atherosclerotic lesions and is considered as a surrogate marker of subclinical atherosclerosis. Numerous studies have demonstrated that both type 1 and type 2 diabetic patients had significantly higher CIMT than control subjects. For example, Järvisalo *et al.* demonstrated that children with type 1 diabetes had increased carotid IMT compared to control subjects based on ultrasound examination [8]. Additionally, Pujia *et al.* observed that type 2 diabetes subjects had larger CIMT compared with control subjects [39]. It is well-established that increased CIMT is associated with cardiovascular risk factors such as metabolic syndrome (MS) and insulin resistance (IR), coronary atherosclerosis, and cardiovascular events [42-47]. Herder *et al.* found that MS was associated with CIMT and progression of CIMT [47]. Subjects with MS have higher levels of CIMT at follow up than those without MS [47], which indicate MS may be involved in the initiation of the process of atherosclerosis. Furthermore, subjects with MS have significantly higher incidence of subclinical carotid atherosclerosis and cardiovascular events than subjects without MS, irrespective of its several definitions [48]. Obesity is closely associated with IR and MS. However, even in non-obese adolescent type 1 diabetes, CIMT is increased and also associated with IR [46]. Thus, IR may be used an important factor reflecting early signs of atherosclerosis in type 1 diabetes. We therefore hypothesise that an increase of CIMT in diabetic patients with ketosis-onset may also result in a higher risk of cardiovascular disease in the future.

Consistent with other studies [33,49-51], we found that some of the traditional risk factors for atherosclerosis were also present in the patients with ketosis-onset diabetes. As expected, age, hypertension, LDL-C, and CIMT were independently associated with the presence of carotid atherosclerosis in the ketosis-onset type 2 diabetes. Therefore, strict control of hypertension and dyslipidaemia is important in order to prevent atherosclerosis in the carotid arteries in diabetic patients with ketosis-onset.

### Limitations

Some limitations need to be considered for this study. The cross-sectional design limits our ability to infer a causal relationship between diabetic ketosis and the occurrence of carotid atherosclerosis. Further research is needed to investigate the long-term effects of diabetic ketosis on atherosclerosis in ketosis-prone diabetes. In addition to small sample size, the present study was also limited by the fact that ketosis-onset diabetic patients were not gender-matched because of the well-recognised predominance of ketosis-onset diabetes in males. Further studies with larger cohorts will be necessary to determine the prevalence of atherosclerosis in ketosis-onset type 2 diabetes. Finally, because of the lack of a type 1 diabetic group, the difference of carotid atherosclerotic lesions between type 1 diabetics and ketosis-onset diabetics could not be compared.

## Conclusions

In conclusion, ketosis-prone type 2 diabetes was common among Chinese patients with new-onset diabetes. The prevalence and risk of carotid atherosclerosis in ketosis-onset diabetics without islet-associated autoantibodies were significantly higher than that of control subjects without diabetes but similar to that of non-ketotic type 2 diabetic subjects. The characteristics of carotid atherosclerotic lesions in ketosis-onset diabetes resemble those of type 2 diabetes. The presence of carotid atherosclerosis was significantly associated with age, hypertension, LDL-C and CIMT in the ketosis-onset diabetes. The present study provides further evidence to support the classification of ketosis-prone diabetes as a subtype of type 2 diabetes rather than idiopathic Type I diabetes in adults.

## Abbreviations

CIMT: Carotid Intima-Media-Thickness; BMI: Body Mass Index; WHR: Waist-to-Hip Ratio; SBP: Systolic Blood Pressure; DBP: Diastolic Blood Pressure; LDL-C: Low-Density Lipoprotein Cholesterol; FPG: Fasting Plasma Glucose; 2-h PPG: 2-h Postprandial Plasma Glucose; GAD: Glutamic Acid Decarboxylase; IA-2: Thyrosin Phosphatase; HbA1c: Glycated Hemoglobin A1C; FCP: Fasting C-Peptide; 2-h PCP: 2-h Postprandial C-Peptide; TG: Total Triglycerides; TC: Total Cholesterol; HDL-C: High-Density Lipoprotein Cholesterol; MS: Metabolic Syndrome; IR: Insulin Resistance.

## Competing interests

The authors declare that they have no competing interests.

## Authors’ contributions

Li LX and Jia WP performed the study design, and supervised this work, and reviewed and edited the manuscript. Li LX, Zhao CC, and Ren Y researched data, and performed statistical analysis and wrote the manuscript. Wu X, Zhang WX, and Zhu JA performed ultrasound examination and data collection and researched data. Lu JX, Tu YF, Li MF, Yu LB, and Bao YQ researched data and reviewed the manuscript. All authors read and approved the final manuscript.

## Supplementary Material

Additional file 1 **Table S1. **Comparison of clinical characteristics in subjects with and without carotid atherosclerosis. Click here for file
