# Supplementary material for: Prevalence and clinical characteristics of carotid atherosclerosis in newly diagnosed patients with ketosis-onset diabetes: a cross-sectional study
Source: Cardiovasc Diabetol. 2013 Jan 16;12:18. doi: 10.1186/1475-2840-12-18 (PMC3583071; doi:10.1186/1475-2840-12-18)
Supplement: Additional file 1 — Table S1. Comparison of clinical characteristics in subjects with and without carotid atherosclerosis. [file 1475-2840-12-18-S1.doc]

**Table S1** Comparison of clinical characteristics in subjects with and without carotid atherosclerosis

|  | Control subjects (n=79) |  |  | Ketosis-onset type 2 diabetes (n=208) |  |  | Non-ketotic type 2 diabetes (n=215) |  |  |
| --- | --- | --- | --- | --- | --- | --- | --- | --- | --- |
| Variables | Without carotid  atherosclerosis (n=67) | With carotid  atherosclerosis (n=12) | P-value | Without carotid  atherosclerosis (n=144) | With carotid  atherosclerosis (n=64) | P-value | Without carotid  atherosclerosis (n=138) | With carotid  atherosclerosis (n=77) | P-value |
| Male (%) | 36 (53.7%) | 10(83.3%) | 0.056 | 108(75.0%) | 45(70.3%) | 0.479 | 77(55.8%) | 45(58.4%) | 0.707 |
| Age (years) | 49.16±6.99 | 52.00±6.24 | 0.183 | 44.49±13.92 | 59.45±12.58 | <0.001 | 45.43±11.27 | 62.06±11.38 | <0.001 |
| Smoking (n, %) | 36(53.7%) | 8(66.7%) | 0.403 | 59(41.0%) | 25(39.9%) | 0.854 | 23(16.7%) | 20(26.0%) | 0.108 |
| Alcohol (n, %) | 37(55.2%) | 7(58.3%) | 0.842 | 26(18.1%) | 11(17.2% | 0.880 | 11(15.3%) | 13(16.9%) | 0.967 |
| Hypertension (%) | 15(22.4%) | 2(16.7%) | 0.657 | 42(29.2%) | 39(60.9%) | <0.001 | 49(35.5%) | 49(63.6%) | <0.001 |
| BMI (kg/m2) | 24.61±3.55 | 24.83±3.40 | 0.843 | 25.26±3.68 | 24.58±3.18 | 0.198 | 25.49±3.76 | 25.00±3.51 | 0.348 |
| WHR | 0.90±0.05 | 0.90±0.04 | 0.680 | 0.92±0.06 | 0.90±0.05 | 0.100 | 0.90±0.05 | 0.92±0.06 | 0.025 |
| Weight category |  |  | 0.893 |  |  | 0.706 |  |  | 0.576 |
| underweight | 0(0.00%) | 0(0.00%) |  | 2(1.4%) | 1(1.6%) |  | 1(0.7%) | 1(1.3%) |  |
| overweight | 19(28.4%) | 4(33.3%) |  | 31(21.5%) | 17(26.6%) |  | 27(19.6%) | 21(27.3%) |  |
| obesity | 27(40.3%) | 5(41.7%) |  | 71(49.3%) | 26(40.6%) |  | 72(52.2%) | 37(48.1%) |  |
| SBP (mmHg) | 127.49±13.76 | 135.00±17.02 | 0.097 | 124.99±14.30 | 131.23±14.88 | 0.005 | 127.49±14.74 | 133.96±19.05 | 0.011 |
| DBP (mmHg) | 83.34±10.44 | 90.50±16.05 | 0.049 | 81.01±10.48 | 81.55±11.66 | 0.741 | 80.72±8.77 | 82.09±10.30 | 0.303 |
| *TG (mmol/l) | 1.43(0.98-2.04) | 1.63(1.38-2.21) | 0.115 | 1.52(0.96-2.42) | 1.40(1.07-2.14) | 0.852 | 1.43(1.08-2.12) | 1.57(1.08-2.41) | 0.372 |
| TC (mmol/l) | 4.94±1.01 | 5.64±1.44 | 0.041 | 4.97±1.24 | 5.15±1.18 | 0.324 | 4.78±1.22 | 4.90±1.24 | 0.511 |
| HDL (mmol/l) | 1.46±0.35 | 1.40±0.25 | 0.561 | 1.05±0.28 | 1.05±0.25 | 0.951 | 1.12±0.24 | 1.10±0.26 | 0.628 |
| LDL (mmol/l) | 3.73±1.19 | 4.09±1.51 | 0.358 | 3.28±1.04 | 3.67±0.96 | 0.012 | 3.12±0.91 | 3.12±1.08 | 0.983 |
| *FPG (mmol/l) | 5.28(4.95-5.65) | 5.52(5.31-5.84) | 0.048 | 10.04(7.94-13.07) | 8.75(6.50-11.16) | 0.013 | 7.52(6.50-9.50) | 7.64(6.17-9.80) | 0.349 |
| *2h PPG (mmol/l) | 5.04(4.45-6.08) | 4.88(4.58-6.10) | 0.875 | 16.42(12.97-20.86) | 17.26(12.14-20.40) | 0.780 | 13.14(10.56-16.45) | 13.17(10.11-16.96) | 0.840 |
| *FCP | —— | —— | —— | 1.23(0.76-1.86) | 1.23(0.66-1.94) | 0.715 | 2.11(1.40-2.96) | 1.84(1.28-2.91) | 0.799 |
| *2h CP | —— | —— | —— | 2.30(1.44-3.65) | 2.35(1.56-3.80) | 0.740 | 4.77(3.14-6.49) | 4.51(3.06-6.22) | 0.862 |
| HA1C (%) | 5.46±0.44 | 5.35±0.37 | 0.419 | 11.78±1.99 | 11.95±2.20 | 0.631 | 9.78±2.39 | 9.86±2.91 | 0.842 |

Values are expressed as the mean±S.D, median with interquartile range, or percentages. * The Mann-Whitney U–test was applied.
